# O007. Self-referred cognitive impairment in migraine patients

**DOI:** 10.1186/1129-2377-16-S1-A149

**Published:** 2015-09-28

**Authors:** Amerigo Costa, Alessandra Sansalone, Aida Squillace, Giuseppe Vescio, Rosario Iannacchero

**Affiliations:** Centre for Headache and Adaptive Disorders, Unit of Neurology, Department of Neuroscience and Sense Organs, Azienda Ospedaliera “Pugliese-Ciaccio”, Catanzaro, Italy; Department of Health Science, Magna Graecia University, Catanzaro, Italy

## Background

Migraine patients often report cognitive impairment, especially regarding memory and attention. There is no consensus about the relationship between migraine and cognitive problems [1]. Aim of our open cross-sectional study was to explore the cognitive performance of migraine patients accessing our Headache Centre and its relationship with demographic, clinical and psychopathological measures.

## Materials and methods

We assigned 30 migraine patients (25 females; 36.63±9.13 mean age) accessing to our Centre from November 2014 to May 2015 to one of three groups according to migraine frequency. Group A patients had no or little chronicity (< 5 headache days/month; n=9); Group B patients had moderate chronicity (> 5 < 10 headache days/month; n=10); Group C patients had severe chronicity (> 10 headache days/month; n=11) [2]. All patients had completed a headache diary, pain Numeric Rating Scale (NRS) and Migraine Disability Assessment (MIDAS) during headache assessment. We measured affective dimensions using Zung Self-Rating Anxiety Scale (SAS), Zung Self-Rating Depression Scale (SDS) and Hypomania Checklist (HCL-32) and we used the Cognitive Failures Questionnaire (CFQ) to quantify cognitive impairment. CFQ measures individual differences in daily cognitive errors, with 25 questions on a Likert-scale relating to everyday mistakes such as the probability of failing to keep a task objective in mind. Higher CFQ scores correspond to a higher cognitive impairment [3]. Using SOFA Statistics 1.4.4 software, we calculated descriptive indicators and Pearson's correlation coefficient (r) between all measures. We compared groups on demographic, clinical, affective and cognitive variables by One-Way analysis of variance (ANOVA). We set p < 0.05 as threshold of statistical significance.

## Results

**Figure 1:**
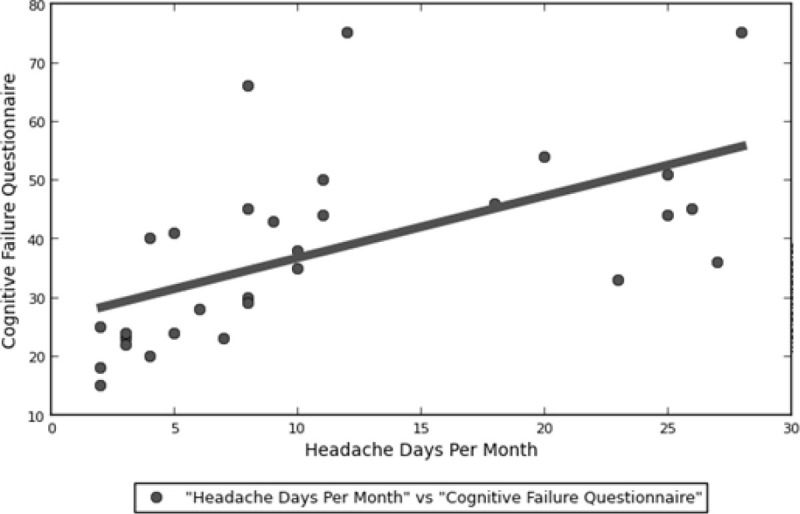
Correlation between migraine chronicity and cognitive self-referred impairment.

CFQ scores were highest (M±DS) among Group C patients (50.27±13.65) followed by Group B (37.83±12.23) and Group A patients (23.63±7.00). ANOVA showed statistically significant difference between groups on CFQ scores (p < 0.01; F = 13.357). CFQ scores positively correlated with migraine frequency (p < 0.001; r = 0.573; fig. 1), MIDAS scores (p < 0.001; r = 0.614), SAS scores (p < 0.001; r = 0.743) and SDS scores (p < 0.05; r = 0.556).

## Conclusions

Migraine patients accessing our Centre report cognitive issues that increase with headache frequency. Such impairment is associated with anxiety and depression levels and contributes to headache-induced disability. Further developments of our study should involve larger groups, include healthy controls and could also investigate the role of medical and psychological headache management in patients’ cognitive performance.

Written informed consent to publish was obtained from the patient(s).
